# Exercise behavior, practice, injury, and symptoms of respiratory tract infection of 502 Brazilian adults during lockdown oscillations in two years (2021–2022) of the COVID-19 pandemic

**DOI:** 10.1186/s13102-023-00701-8

**Published:** 2023-08-01

**Authors:** Lucas Guilherme Oliveira da Silva, Tatiane Silva de Souza, Camila Réquia Silva, Flávia Figueiredo Freua, Lucas Barqueiro Medeiro da Silva, Yara Juliano, Luiz Henrique Silva Nali, Luiz Carlos Hespanhol, Ana Paula Ribeiro

**Affiliations:** 1 Biomechanics and Musculoskeletal Rehabilitation Laboratory, Health Science Post-Graduate Department, School of Medicine, University Santo Amaro, R. Professor Enéas de Siqueira Neto, 340, Campus I, São Paulo, SP 04829-900 Brazil; 2 Physical Therapy Post-Graduate Department, University City of Sao Paulo, São Paulo, Brazil; 3 Physical Therapy, Speech and Occupational Therapy Department, School of Medicine, University of Sao Paulo, Sao Paulo, Brazil

**Keywords:** COVID-19, Exercise, Injuries, Pandemic, Symptoms

## Abstract

**Background:**

In the period between 2020 and 2023, during the COVID-19 (coronavirus disease 2019) pandemic, many countries released their restriction measures so that individuals were able to begin practicing physical exercises and outdoor sports again. The purpose of the current study was to evaluate the physical exercise behavior, symptoms of respiratory tract infection, and training practice, as well as aspects of pain and injuries in the lower limbs of adults during periods of lockdown oscillations in the two years of the COVID-19 pandemic in Brazil.

**Methods:**

Cross-sectional study. Participants: A total of 502 adults were evaluated during two consecutive years of the COVID-19 pandemic, corresponding to the years 2021 and 2022. A virtual questionnaire was applied using the Google Forms platform through a link, or a Quick Response Code available in social media environments. The variables collected were: anthropometric characteristics, presence of comorbidities, clinical history for the diagnosis of COVID-19, and behavior related to physical exercise practices, divided into five topics: (1) physical exercise habits; (2) symptoms and health care utilization; (3) habit of practicing physical exercise in relation to the prevention of COVID-19; (4) preventive measures for COVID-19; and (5) feelings and reasons for practicing exercises.

**Results:**

A total 79.0% of the participants returned to the practice of physical exercise after a period of social isolation due to COVID-19, with running (30.0%) and muscle strength training (50.0%) being the most prevalent modalities, in which 62.0% of practitioners carried out the activity individually, without any professional or technical monitoring. With regard to physical preparation, 61.0% reported performing pre-training stretching, 64.0% associated with muscular resistance training. Of these, 89% did not report current injuries or pain symptoms when returning to exercise (69.0%). Total of 60.5% reported experiencing respiratory tract symptoms of COVID-19 and seeking a consultation with a doctor, and 61.0% performed diagnostic test, with RT-PCR (Real time-polymerase chain reaction) being the most common test. Of those tested, 55.0% were positive for COVID-19, without the need for hospitalization (95.0%). The most commonly used measures for the prevention of COVID-19 were the fabric or surgical mask. The predominant feeling in the pandemic was anxiety (50.5%) and the reasons for practicing sports were: physical conditioning (30.9%), a feeling of pleasure (21.3%), and weight loss (20.3%).

**Conclusion:**

After two years of the COVID-19 pandemic (2021–2022), with periods of lockdown, there were low reports of injuries and pain symptoms after exercising on the return to physical exercise practices of running and strength training. However, the restrictions negatively affected the exercise behavior due to respiratory tract symptoms of COVID-19 and a reduction in training intensity, performed without any professional or technical supervision. The participants reported the use of a fabric or surgical mask for the prevention of COVID-19, and an increased feeling of anxiety. The reasons given for practicing physical exercise were physical conditioning, a feeling of pleasure, and weight loss.

**Supplementary Information:**

The online version contains supplementary material available at 10.1186/s13102-023-00701-8.

## Background

Coronavirus disease 2019 (COVID-19) is an infectious disease caused by severe acute respiratory syndrome coronavirus 2 (SARS-CoV-2), which was discovered in December 2019 in Wuhan [1]. Poor dietary habits and physical inactivity are characterized by chronic and high inflammation [2]. It should be noted that moderate levels of physical exercise improve immunity and could provide an immuno-protective effect [1, 2].

Physical exercise through running is one of the most popular in the world, which can be explained by the fact it is easily accessible, requiring minimal equipment and structure for its practice [1]. Physical exercise has been associated with a reduction in the risk of chronic and cardiovascular diseases [2] and an improvement in mental health [3], making this form of exercise an attractive health behavior for the general population. Furthermore, many runners choose to train in groups, clubs, or teams, thus introducing an important social aspect to their practice [3, 4]. In March 2020, athlete training was compromised for numerous reasons, due to repetitive periods of local/national lockdowns (with social distancing, movement restrictions, and facility closures), i.e., termed lockdown oscillations in the current study [4, 5]. Closures of athlete’s training facilities hindered athlete’s access to proper training and their multidisciplinary teams (e.g., coaches, medical/health staff) [5].

The practice and return to physical exercise in periods of the COVID-19 pandemic in Brazil were essential. However, as of January 2023, data reveal that the pandemic is still continuing in the country, with 36,477,214 confirmed cases of the disease, with a mortality rate of 694,779, corresponding to an incidence of 17,357.9/100,000 inhabitants and a mortality rate of 330.6/100,000 inhabitants [4]. In light of the mortality rates caused by the COVID-19 pandemic and the literature indicating that physical activity can improve immunity [5, 6], thereby influencing the effects of COVID-19 complications, it is necessary to understand physical exercise practices (what have been done) in “confined situations” on a long-term basis, i.e., the two-year period (2021–2022) of the pandemic, in order to improve the strategy of return and continuity in physical exercise during pandemic situations”. Moreover, even among “more active people” or athletes, there were variations in their physical activities [5]. For examples, some individuals, particularly those who participated in team-based sports, were adversely affected by the “confinement situations” to the extent that they required remote guidance to perform physical exercises [5].

Running is one of the most commonly performed types of exercise, however despite the advantages of exercise in terms of reducing sedentary behavior and enhancing physical health, lower limb injuries are common (19.4–79.2%) [5–7]. Another important point is the acute coronary syndrome and myocarditis after COVID-19 diagnosis [8], which have a negative influence on the practice of physical exercise [9]. The COVID-19 pandemic has imposed a unique and overarching demand across the globe, leading to government directives including self-isolation and/or lockdowns, in order to mitigate the spread or propagation of this deadly virus. These restrictions provoke decreases in the practice of physical exercise at home, after work, and in leisure [10–12], with COVID-19 home confinement resulting in significantly negative alterations in physical-activity levels [10, 11]. In addition, lockdown compromised training-related practices, especially in lower-level athletes, with reductions in training specificity, intensity, frequency, and duration. Furthermore, many athletes showed a need for assistance with training and tools to cope with anxiety [12].

These recurrent transitory moments of quarantine periods have resulted in a significant impact not only on the physical and mental health of individuals, but also on the economy of society as a whole [13]. In addition, widespread changes in this period caused the closure of gyms and fitness training facilities, the closure of formal and informal group activities, and restrictions on parks and trails, which disrupted community norms of long-distance running [14].

The COVID-19 pandemic also led to many races being cancelled or postponed, which inevitably resulted in training changes for competitive, amateur, and recreational athletes [15, 16]. According to Washif et al. [11], in 2022, the COVID-19-related lockdowns reduced athletic training specificity, intensity, frequency, and duration, with notable differences in athlete classifications. However, there is currently no information available on how the pandemic has influenced training behaviors in Brazilian physical exercise practitioners, particularly with regard to practice volume, intensity, training surfaces, injuries and reasons for engaging in physical or sporting activities.

Previous epidemiological research studies found high incidence rates of injuries in the population of runners, reaching up to 90% of those who practice the sport [7]. In these athletes, the majority of injuries are located in the lower extremities, especially, patellofemoral pain syndrome, Achilles tendinopathy, and plantar fasciitis [7, 16–18]. The majority of injuries are attributed mainly to poor or inappropriate exercise prescription [19], such as sudden increases in the volume and intensity of running, generating large joint overloads [19, 20]. Recent studies have shown that the pandemic influenced the behavior of runners, with a reduction in the volume and intensity of training, less motivation to run, and less supervision by a professional or technical trainer [14, 15]. In addition, an increased risk of injury was observed, with the rate of injuries increasing by 1.4 times during the pandemic, especially in the knees and feet [14]. Another study evaluating the return to outdoor running, after a period of quarantine, revealed that recreational and amateur runners reduced their training load and started to practice without professional supervision during the confinement period, in relation to more advanced runners with greater experience. This reduction led to decreased physical performance after a period of quarantine, with a greater risk of injuries [21].

COVID-19 presents unique external pressure on the physical activity and running community and is likely to affect the occurrence of injuries in this population [20]. According to Jafarnezhadgero et al. [20], in 2022, COVID-19 infections compromised cardiorespiratory fitness and perceived effort, which may lead to a sub-optimal running style (longer foot contact time with reduced peak propulsion forces). In this context, it is of great importance to understand physical exercise training behaviors and reasons for engaging, considering periods of lockdown in adults. A recent study, in 2023, reported that lockdown compromised training-related practices, especially in lower-level athletes, who require assistance with training and tools to cope with anxiety, tailored to the needs of individual countries. Thus, the purpose of the current study was to evaluate the physical exercise behavior, symptoms of respiratory tract infection, training practice, and aspects of pain and injuries in the lower limbs of adults during periods of lockdown oscillations in two years of the COVID-19 pandemic in Brazil. We hypothesized that periods of lockdown during the COVID-19 pandemic had an effect on physical exercise behavior with increased respiratory symptoms, post-training pain and lower limb injuries.

## Methods

### Study design and sample selection

**Fig. 1:**
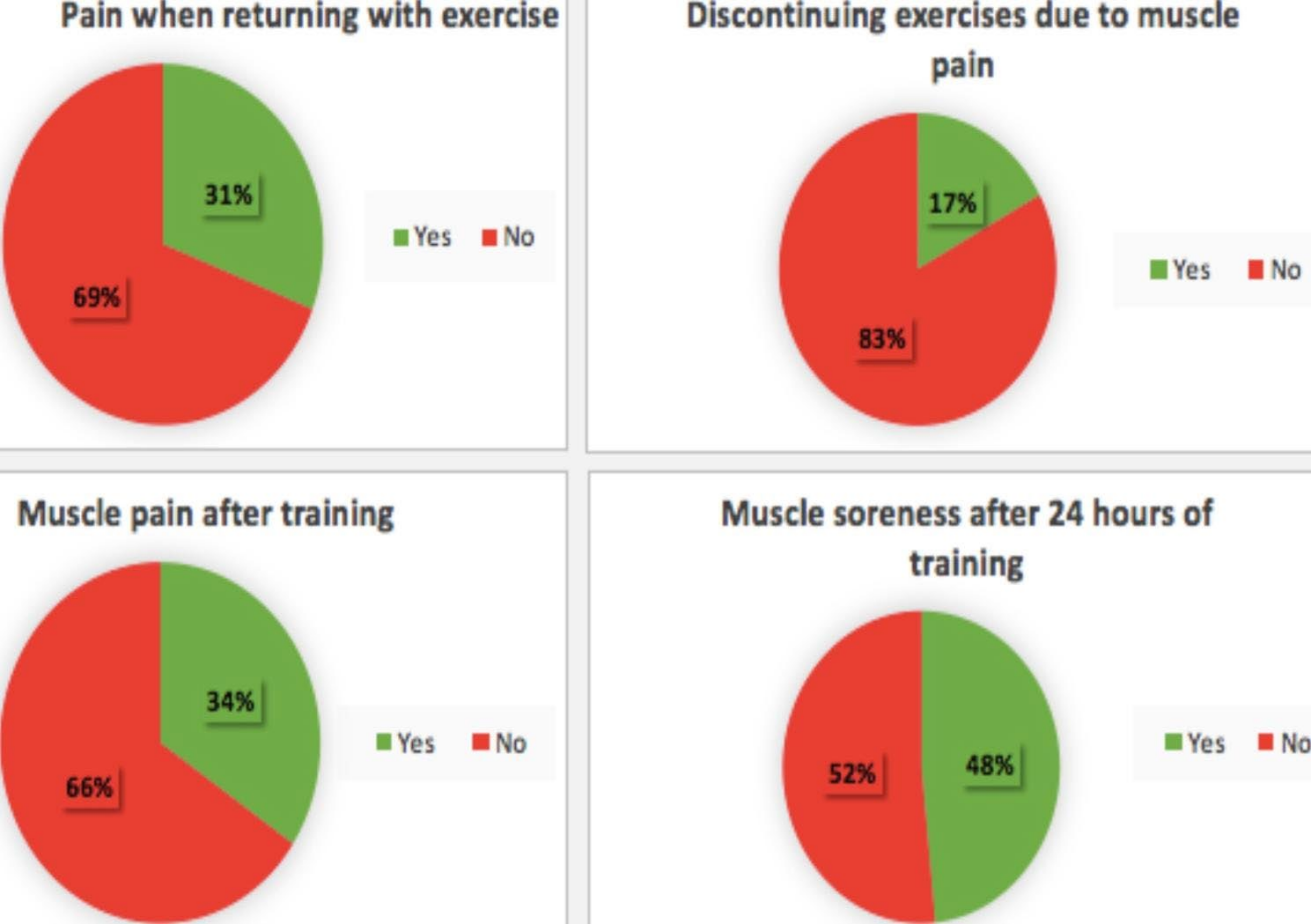
Percentage of pain symptoms before, during, and after physical exercise training performed by young adults during the two years of the COVID-19 pandemic (coronavirus disease 2019)

This research is a cross-sectional study, in which adults who practice physical exercise, residing in different states and cities in Brazil were recruited through convenience sampling, between the years of January 2021 to December 2022 (two consecutive years of the COVID-19 pandemic) (Fig. 1).

The study protocol was reviewed and approved by the Departmental Research Committee of the University Santo Amaro-UNISA (registration number: 4.943.364), in accordance with the Helsinki Declaration and relevant guidelines and regulations. Prior to participation, all participants electronically signed the free and informed consent form, prepared in accordance with resolution 466/12 of the National Health Council.

The eligibility criteria for this study were: physical exercise practitioners aged 18–55 years, training at least 1–2 times a week, outdoor or indoor training after the COVID-19 quarantine period in São Paulo, Brazil, and having practiced physical exercise for more than 1 year. Exclusion criteria were: not practicing physical exercise, or presenting a diagnosis of neurological, cognitive, or mental diseases which could make it impossible to understand to answer the questionnaire. For data cleaning process, incomplete or incongruent responses on the questionnaire or duplicate response were disregarded, as well as present the diagnosis of diseases and an absence of responses to any item of the questionnaire.

### Clinical diagnosis of COVID-19 and evaluation protocol

The epidemiological characteristics of the physical exercise practitioners were collected during oscillatory periods of quarantine (social isolation) arising from the COVID-19 pandemic. For participants who responded as positive for the clinical diagnosis of COVID-19, a clinical diagnosis confirmed by laboratory or imaging tests was accepted, including:

- **Laboratory diagnosis** performed either by molecular biology or serology tests. Molecular biology tests: enable identification of the presence of the genetic material (RNA) of the SARS-CoV-2 virus in respiratory secretion samples, using RT-PCR (Real time-polymerase chain reaction) methodologies and reverse transcriptase loop-mediated isothermal amplification. Serology tests: detect immunoglobulin M (IgM), immunoglobulin A (IgA), and/or immunoglobulin G (IgG) antibodies produced by the individual’s immune response to the SARS-CoV-2 virus, being able to diagnose active or previous disease. The main methodologies are: Enzyme-Linked Immunosorbent Assay, Chemiluminescence Immunoassay, and Electrochemiluminescence Immunoassay.
- **Diagnostic imaging**: performed by high-resolution Computed Tomography, with the tomographic alterations that are compatible with a case of COVID-19: - peripheral ground-glass opacity, bilateral, with or without consolidation or visible intralobular lines (“paving”); - Multifocal ground-glass opacity of rounded morphology with or without consolidation or visible intralobular lines (“paving”); and Reverse halo sign or other findings of organizing pneumonia (seen later in the disease) [8].

### Evaluation protocol

For the evaluation of the participants, virtual questionnaires were applied, online, using the Google Forms platform, guaranteeing the anonymity and confidentiality of the participants at all times. Data were collected during the COVID-19 pandemic, corresponding to the months of April and May 2021 and January to July 2022, from different municipalities in the state of São Paulo, Brazil. The questionnaires were delivered through a Google Forms link or Quick Response Code available in social media environments, such as: Facebook, Instagram, and WhatsApp groups. In addition, the link to access the questionnaire and the term of acceptance for participating in the research were also made available on websites corresponding to running clubs or associations in the city of São Paulo.

An initial questionnaire was applied, containing information on anthropometric characteristics (sex, age, weight, height and body mass index-BMI), the presence of comorbidities, such as: arterial hypertension and diabetes mellitus, and clinical characteristics of the history of a diagnosis of COVID-19. When positive, data were collected on if there was a need for hospitalization and the period of hospitalization [21]. Subsequently, a questionnaire was applied on the practice of physical exercises during the COVID-19 pandemic, still in the online and self-reporting form, following the criteria proposed by Cloosterman et al., (2021) [22] and Mosqueira-Ourens et al., (2021) [21], aiming to investigate the behavior and habits of physical exercise practitioners during the COVID-19 pandemic. This questionnaire was translated by a native speaker of the Portuguese language and all questions were adapted for understanding in the native language (appendix-questionnaire), since the study of validity of the questions was carried out by the same group of researchers. The tool consists of five assessment items:

1. Physical exercise habits: physical exercise habits during quarantine (period of social isolation) were assessed by asking whether participants continued to exercise outdoors (yes/no) and, if so, their average weekly training frequency, times of exercising, distance and speed, and training kilometers, when relevant (average per week over the previous 7 weeks).
2. Symptoms and health care utilization: participants were asked if they had experienced symptoms of COVID-19 (yes/no) in the previous 7 weeks, including runny nose, sore throat, fever, dry or productive cough, dyspnoea during rest or exertion, myalgia, headache, chest pain, diarrhea, nausea or vomiting, eye infection, dysosmia, and fatigue. If so, participants were asked whether they had seen a general practitioner due to their symptoms (yes/no), whether they had been tested for COVID-19 (yes/no), which test had been performed and the result of this test (positive / negative with briefly clarify each COVID-19 diagnostic test), and whether they were hospitalized due to COVID-19 (yes / no). If the participant reported being hospitalized, information was obtained on the number of days and admission to intensive care.
3. Behavior of physical exercise in relation to the prevention of COVID-19: physical exercise behavior during periods characterized by quarantine (social isolation) and after COVID-19 quarantine were evaluated, including the type of training (resistance / interval / specific exercises, in which short definitions were presented after the question about each type of training for better understanding of the participant), your pace of training - based on the metabolic equivalent for task - MET (physical effort to perform the exercise), considering light training: 2 METs; moderate training: 3–6 METs and intense training: 7 or more METs), training with a partner or family member (yes / no), whether a physical distance of 1.5 m was maintained during training (yes / no), and associated injuries (simple definition of each injury considered). Another evaluation point concerned interval and dichotomized training in more or less than 50% of the training.
4. Preventive measures for COVID-19: participants were asked whether they followed measures to prevent the transmission of COVID-19. The measures questioned were meticulous hand hygiene, avoiding touching the face, eyes, and mouth, wearing a face mask, physical distancing, avoiding unnecessary travel, and avoiding group gatherings. In addition, they were asked about the use of a mask throughout the training practice and their discomfort, with scores ranging from 0 to 10, with zero being the worst possible discomfort. Another point questioned was the habit of practicing physical exercise at home during quarantine (weekly frequency and intensity).
5. Feelings and reasons for practicing physical exercise during COVID-19: Participants, in this item, were asked about their feelings and emotions during the COVID-19 pandemic. The feelings questioned were: sadness, sleep disturbance, tranquility, anxiety, and others. In addition, the reasons for adherence to physical exercise were questioned, namely: pleasure, weight loss, leisure, physical conditioning, stress relief, and others [21, 22].

### Statistical analysis

All statistical analyses were performed using SPSS version 24 (IBM, Chicago, IL, USA). The normality of the data was verified using the Shapiro–Wilk test. Descriptive analysis is reported as number and percentage, as well as the mean and standard deviation of some quantitative variables. For comparisons of anthropometric characteristics between sexes, the independent student-t test was used. For all analyses, significant differences were considered when p < 0.05.

## Results

The questionnaire was sent to all participants and 502 were returned fully completed, with all responses. The majority of respondents were female, 53.0%, with a mean age of 34.5 ± 10.2 years, body mass of 66.5 ± 11.3 kg, and height of 1.62 ± 6.7 m; men accounted for 47.0%, with a mean age of 32.9 ± 11.5 years, body mass 81.2 ± 17.5 kg, and height 1.76 ± 7.2 m, with no statistical differences for any of the anthropometric variables (p > 0.005), when compared between sexes.

The state with the highest prevalence of responses was in the city of São Paulo. Most participants did not report the presence of chronic diseases (comorbidity), such as arterial hypertension and diabetes mellitus, and were non-smokers. The consumption of alcoholic beverages predominated, with frequencies of 1 to 3 times a week, the most frequent consumption being of beer and wine, as observed in Table 1.

**Table 1:** Profile of anthropometric characteristics and comorbidities of young adults practicing physical exercise during the two consecutive years of the COVID-19 pandemic (coronavirus disease 2019)

Characteristics	Variables	N	%
Sex	Female	267	53%
	Male	235	47%
	**Total**	**502**	**100%**
State	São Paulo	452	90.0%
	Other municipalities in the state of São Paulo	50	9.6%
	**Total**	**502**	**100%**
Do you have a chronic illness?	Yes	74	15%
	No	428	85%
	Total	502	
Do you consume any alcoholic beverages socially?	Yes	339	68%
	No	163	32%
	**Total**	**502**	**100%**
How often weekly?	1–3	332	98%
	4–6	7	2%
	**Total**	**339**	**100%**
Which beverage?	Beer	188	55%
	Wine	104	31%
	Whisky	24	7%
	Gin	18	5%
	Vodka	5	1%
	**Total**	**339**	**100%**
Do you consider yourself a smoker?	Yes	23	5%
	No	479	95%
	**Total**	**502**	**100%**

Table 2 presents the category and habits of the practice of physical exercise by the adults participating in the research. The majority were characterized in the physical exercise practitioner categories of amateur (40.0%) and professional (39.0%). Two physical exercises were the most prevalent, running, with 30.0% and muscle strength training (gym) with 50.0%. In addition, 55.0% did not change the frequency and intensity of training during the week. Furthermore, 59.0% reported practicing interval training and 47% aerobic activity. The majority (80.0%) reported not being affiliated with clubs or associations for training, with 62.0% not having a training program guided by a specialized professional.

**Table 2:** Profile of the categories and habits of the practice of physical exercise evaluated during the two consecutive years of the COVID-19 pandemic (coronavirus disease 2019) in young adults

Characteristics	Variables	N	%
What type of physical activity practitioner do you classify yourself as?	Beginner Practitioner	102	20%
	Amateur Practitioner	203	40%
	Advanced Practitioner	197	39%
	**Total**	**502**	**100%**
What type of exercise do you practice?	Gym	252	50%
	Running	153	30%
	Football	33	7%
	Cycling	30	6%
	Volleyball	10	2%
	Fight modality	9	2%
	Tennis	4	1%
	Handball	3	1%
	Swimming	6	1%
	Basketball	2	0%
	**Total**	**502**	**100%**
Do you alternate the intensity of physical activity during the week?	Yes	224	45%
	No	278	55%
	**Total**	**502**	**100%**
Do you perform interval training?	Yes	297	59%
	No	205	41%
	**Total**	**502**	**100%**
If the previous answer was yes, in what way the exercise in interval training?	Aerobic activity	140	47%
	Walking	84	28%
	Sport activity	73	25%
	**Total**	**297**	**100%**
Are you affiliated with any club or coaching advisor?	Yes	102	20%
	No	400	80%
	**Total**	**502**	**100%**
Do you have a training program (schedule)?	Yes	192	38%
	No	310	62%
	**Total**	**502**	**100%**
What is the ground surface used in your training?	Asphalt	334	67%
	Tartan	102	20%
	Earth	33	7%
	Grass	30	6%
	Sand	3	1%
	**Total**	**502**	**100%**
What is your predominant training location?	Gym (other)	189	38%
	Road	167	33%
	Inside the home	48	10%
	Park	45	9%
	Club	31	6%
	Condominium	20	4%
	Farm	2	0%
	**Total**	**502**	**100%**
Do you warm up with pre-training stretching?	Yes	307	61%
	No	195	39%
	**Total**	**502**	**100%**
Do you cool down with post-training stretching?	Yes	225	45%
	No	277	55%
	**Total**	**502**	**100%**
Do you perform muscle resistance training?	Yes	321	64%
	No	181	36%
	**Total**	**502**	**100%**
For your training practice, do you know if you have vitamin D deficiency?	Yes	101	20%
	No	164	33%
	I don’t know	237	47%
	**Total**	**502**	**100%**
What type of footwear do you use most during physical activity?	Footwear with flat and flexible soles	160	31.8%
	Footwear with a higher sole at the heel	157	31.3%
	Other	54	11%
	Footwear with insole	50	9.1%
	Neutral footwear	47	9%
	Footwear with flat and rigid soles	14	3%
	Footwear with carbon plate	9	2%
	Footwear for supination	5	1%
	Footwear for pronation	6	1%
	**Total**	**502**	**100%**
Did you receive guidance on your shoes for physical activity?	Yes	178	35%
	No	324	65%
	**Total**	**502**	**100%**
If the previous answer is yes, which professional guided you?	Physical educator	66	37.0%
	Physiotherapist	37	20.7%
	Coach	33	18.5%
	Other	30	17%
	Doctor	12	7%
	**Total**	**178**	**100%**
Do you use any bandages in training practice?	Yes	39	8%
	No	463	92%
	**Total**	**502**	**100%**

The most frequent training surface was asphalt, reported by 67.0% of the participants, with a predominance of the street (33.0%) and gym (38.0%) for the training locations. With regard to preparation for the practice of physical exercise, 61.0% reported performing pre-training stretching, associated with muscular resistance training (64.0%). Regarding the characteristics of the training shoes, the prevalent footwear choices were traditional (31.3%) and minimalist (31.8%), without receiving guidance from a professional for use during physical exercise practice. Of the participants who received guidance on sports shoes (35.0%), the most frequent professionals providing guidance were physical educators (37.0%) and physiotherapists (20.7%). A total of 92.0% reported not using a functional bandage during training, and 47.0% reported not knowing if they had vitamin D deficiency for the practice of exercises, as observed in Table 2.

Table 3 shows that, predominantly, the participants did not mention inflammatory injuries in the hips and knees, or pain symptoms. A small percentage reported symptoms of medial knee pain (24.0%) and foot pain (14.0%) in the heel region.

**Table 3:** Profile of injuries and pain symptoms in the practice of physical exercise evaluated during the two consecutive years of the COVID-19 pandemic (coronavirus disease 2019) in young adults

Characteristics	Variables	N	%
Do you have a clinical diagnosis of hip osteoarthritis?	Yes	7	1.4%
	No	495	98.6%
	**Total**	**502**	**100%**
Do you have a clinical diagnosis of knee osteoarthritis?	Yes	40	8%
	No	462	92%
	**Total**	**502**	**100%**
Do you currently have pain in your feet?	Yes	70	14%
	No	432	86%
	**Total**	**502**	**100%**
Do you feel more pain in:	Right foot	47	67%
	Left foot	23	33%
	**Total**	**70**	**100%**
What is the most predominant location of pain in the feet?	Hindfoot	33	47%
	Midfoot	22	31%
	Forefoot	15	21%
	**Total**	**70**	**100%**
Do you currently have knee pain?	Yes	120	24%
	No	382	76%
	**Total**	**502**	**100%**
Do you feel more pain in:	Right knee	65	54%
	Left knee	55	46%
	**Total**	**120**	**100%**
What is the most predominant location of pain in the knees?	Anterior	34	28%
	Medial	44	37%
	Lateral	25	21%
	Posterior	17	14%
	**Total**	**120**	**100%**

In Table 4, it can be seen that 56.0% of participants reported a moderate training pace. The most prevalent locations for training during quarantine were the street (32.0%) and the home (39.0%). The period of preference for the practice of training was the morning (44.0%) with the performance of more than one exercise practice in 67.0% of the participants, and a greater prevalence of functional training (28.0%) and muscle force training (22.0%). The majority of participants reported that they did not perform sports (58.0%) during the quarantine periods, however, street running was prevalent in 52.0%. Of these, 67.0% reported not having the supervision of a health professional for guidance. The training was performed individually in 80.0%, with 92.0%following the preventive measures for COVID-19. Fabric (45.0%) or surgical (33.0%) masks were the most commonly reported, and 84.0% maintained social distancing during training practice.

**Table 4:** Profile of the behavior of physical exercise during quarantine and prevention measures for COVID-19 in the period of two consecutive years of the pandemic (coronavirus disease 2019) in young adults

Characteristics	Variables	N	%
What is your pace of training?	Moderate	282	56%
	Light	148	29%
	Intense	72	14%
	**Total**	**502**	**100%**
What was the predominant location of your training practice during quarantine?	Inside the home	197	39%
	Road	162	32%
	Gym (other)	81	16%
	Condominium	34	7%
	Park	16	3%
	Club	8	2%
	Farm	4	1%
	**Total**	**502**	**100%**
What is your typical training start time?	Morning	220	44%
	Night	144	29%
	Afternoon	131	26%
	Early morning	7	1%
	**Total**	**502**	**100%**
Did you practice more than one physical activity during isolation?	Yes	338	67%
	No	164	33%
	**Total**	**502**	**100%**
What other physical activity did you practice?	Functional	95	28%
	Weight training	74	22%
	Solo walking	72	21%
	Other	52	15%
	Cycling	33	10%
	Treadmill walking	12	4%
	**Total**	**338**	**100%**
Did you practice any other sports modality during social isolation?	Yes	213	42%
	No	289	58%
	**Total**	**502**	**100%**
If the previous answer was yes, which sport?	Running	111	52%
	Other	39	18%
	Cycling	18	8%
	Football	17	8%
	Fight modality	14	7%
	Volleyball	5	2%
	Swimming	4	2%
	Basketball	3	1%
	Handball	1	0%
	Tennis	1	0%
	**Total**	**213**	**100%**
Was your training supervised by a professional?	Yes	167	33%
	No	335	67%
	**Total**	**502**	**100%**
If the previous answer was yes, how was it supervised?	Person	88	53%
	Virtual	79	47%
	**Total**	**167**	**100%**
How was your training performed?	Individually	401	80%
	In group	101	20%
	**Total**	**502**	**100%**
Did you follow preventive measures against the coronavirus?	Yes	462	92%
	No	40	8%
	**Total**	**502**	**100%**
Did you use a protective mask during physical activity?	Yes	367	73%
	No	135	27%
	**Total**	**502**	**100%**
If the previous answer was yes, which type of mask did you use predominantly?	Fabric	166	45%
	Surgical	122	33%
	N95	41	11%
	Other	23	6%
	PFF2	11	3%
	With a valve	4	1%
	**Total**	**367**	**100%**
Did you maintain social distancing (1.5 m) during physical activity?	Yes	424	84%
	No	78	16%
	**Total**	**502**	**100%**

Table 5 shows that participants did not report associated injuries after returning to physical exercise. Of the 17.0% who reported injuries, the most prevalent was patellofemoral syndrome, with 35.0% of the reports, which limited the functional activity of walking in 35.0% of the cases. In addition, the participants did not mention current injuries (89.0%) or pain symptoms when returning to practice exercise (69.0%), or interruption of the exercises due to pain (83.0%). Another important point was that they also did not report pain immediately after training (66.0%) and 24 h after their exercise practice (52.0%). Of the participants who reported pain symptoms, pain immediately after training, and 24 h after its completion were prevalent, as shown in Fig. 1.

**Table 5:** Profile of injuries in the previous 12 months and current injuries associated pain symptom on return to physical exercise after period of two consecutive years of the COVID-19 pandemic (coronavirus disease 2019) in young adults

Injuries (previous 12 months)	Variables	N	%
Have you had an injury in the last 12 months?	Yes	84	17%
	No	418	83%
	**Total**	**502**	**100%**
What was the clinical diagnosis of the injury from the last six months or currently?	Patellofemoral syndrome	29	35%
	Achilles Tendinitis	15	18%
	Plantar fasciitis	13	15%
	Lumbosacral syndrome	11	13%
	Iliotibial syndrome	10	12%
	Tibial stress fracture	5	6%
	Femoroacetabular Syndrome	1	1%
	**Total**	**84**	**100%**
What was the location of this injury?	Knee	31	37%
	Feet	14	17%
	Ankle	13	15%
	Lower back region	11	13%
	Back of thigh	6	7%
	Calf	6	7%
	Front of thigh	1	1%
	Buttocks	1	1%
	Toes	1	1%
	**Total**	**84**	**100%**
Do you feel pain when you:	Walk	58	35%
	Squat	47	28%
	Go down the stairs	18	11%
	Perform leisure activity	18	11%
	Climb stairs	18	11%
	Stand-up from a sitting position	7	4%
	**Total**	**166**	**100%**
Do you currently have persistent pain or physical impairment?	Yes	78	16%
	No	424	84%
	**Total**	**502**	**100%**
Do you stop running because of pain?	Yes	104	21%
	No	398	79%
	**Total**	**502**	**100%**
**Injuries (current)**	**Variables**	**N**	**%**
Do you have a current injury?	Yes	56	11%
	No	446	89%
	**Total**	**502**	**100%**
Did you have pain when returning to exercises?	Yes	155	31%
	No	347	69%
	**Total**	**502**	**100%**
Have you interrupted practice because of pain?	Yes	86	17%
	No	416	83%
	**Total**	**502**	**100%**
Do you have pain after training?	Yes	173	34%
	No	329	66%
	**Total**	**502**	**100%**
Do you have pain 24 h after training?	Yes	243	48%
	No	258	52%
	**Total**	**502**	**100%**

Table 6 presents the high prevalence of symptoms of COVID-19, in 60.5% of physical exercise practitioners. The majority reported consulting a doctor (60.5%) and having a diagnostic test, with the PCR test (61%) being the most prevalent test for confirming the diagnosis of COVID-19. Positivity was found in 55% of the 304 participants. The vast majority of participants did not require hospitalization (95.0%) or assistance in the intensive care unit-ICU (75.0%). Regarding recent contagion with other people contaminated by COVID-19, the majority reported no contact. In addition, a high prevalence of feelings of anxiety was reported, with 50.5% of cases during the two years of the pandemic. The prevalent reason for practicing physical exercise was for physical conditioning in 30.9%, followed by a feeling of pleasure with exercise in 21.3% and weight loss in 20.3% of the participants, as shown in Table 6.

**Table 6:** Profile of signs and symptoms, as well as the diagnosis of COVID-19 (coronavirus disease 2019), consequences for hospitalization and feelings and emotions in young adults who practice physical exercise in the period of two consecutive years of the COVID-19 pandemic

COVID-19 Pandemic	Variables	N	%
Did you have symptoms of COVID-19?	Yes	304	60.5%
	No	198	39.4%
	**Total**	**502**	**100%**
Did you consult a doctor?	Yes	304	60.5%
	No	45	8.9%
	**Total**	**249**	**100%**
Did you test for COVID-19?	Yes	304	61%
	No	198	39%
	**Total**	**502**	**100%**
What test was performed?	PCR (Real time-polimerase chain reaction)	230	76%
	pharmacy test	48	16%
	Serology	22	7%
	Computed tomography	4	1%
	**Total**	**304**	**100%**
Test result	Positive	166	55%
	Negative	138	45%
	**Total**	**304**	**100%**
If you had COVID-19, did you need to be hospitalized?	Yes	8	5%
	No	158	95%
	**Total**	**166**	**100%**
Did you need to go to the intensive care unit (ICU)?	Yes	2	25%
	No	6	75%
	**Total**	**8**	**100%**
Have you had contact with COVID-19 in the last 6 months?	Yes	203	40%
	No	299	60%
	**Total**	**502**	**100%**
**Feelings and emotions**	**Variables**	**N**	**%**
Feelings and emotions	Anxious	254	50.6%
	Calm	98	19.5%
	Sadness	73	14.5%
	Sleep disturbance	47	9.4%
	Other	30	6%
	**Total**	**502**	**100%**
Reasons for practicing exercises	Physical conditioning	155	30.9%
	Pleasure	107	21.3%
	Weight loss	102	20.3%
	Stress relief	80	15.9%
	Other	34	6.8%
	Leisure	24	4.8%
	**Total**	**502**	**100%**

## Discussion

During two years of the COVID-19 pandemic (2021–2022), with periods of lockdown, 79.0% of the participants returned to the practice of physical exercise, with running and strength training being the most prevalent modalities, without any professional or technical monitoring. With regard to physical preparation, 61.0% reported performing pre-training stretching associated with muscular resistance training. Of these, 89% did not report current injuries or pain symptoms when returning to exercise and 60.5% reported experiencing respiratory tract symptoms of COVID-19 with a diagnostic test by PCR, of which 55.0% were positive for disease, without the need for hospitalization (95.0%). The measure used for the prevention of COVID-19 was wearing a fabric or surgical mask. The predominant feeling in the pandemic was anxiety and the reasons for practicing sports were: physical conditioning, a feeling of pleasure, and weight loss.

According to evidence, the period of lockdown from the COVID-19 pandemic reduced sleep quality and increased insomnia in elite athletes, primarily associated with longer sleep onset latency and later bedtime, preferred time of day to train, and daytime napping [23]. Lockdown-mediated circadian disruption had more deleterious effects on the sleep quality of individual-sport athletes compared with team-sport athletes [23]. Still in this rationale, a study with Canadian adults observed a prevalence of females in the practice of physical activity during the pandemic and the results showed that 40.5% of inactive adults became less active, while only 22.4% of active adults became less active [24]. According to Lesser and Nienhuis (2020) [24], inactive participants who spent more time involved in outdoor physical activities reported less anxiety. The differential of the present study was to understand the practice of physical exercise in Brazilian adults during the two consecutive years of the COVID-19 pandemic. The results showed that habits of physical exercise practice were maintained for running and strength training. According to Washif et al. [5], during lockdown the perceived training intensity was reduced by 29–41%, depending on sport (largest decline: 38% in team sports, unaffected by sex), but some athletes (range: 7-49%) maintained their training intensity for strength and endurance.

Interestingly, weekly alcohol consumption increased in our cohort during the pandemic, with beer and wine consumption being the most prevalent. This is not in line with other findings on the negative associations of alcohol consumption during the COVID-19 pandemic [25], but is in agreement with the study by Salman et al., (2021) [26], who also observed an increase in alcohol consumption during this period. Although this could be due to the specific demographic characteristics of our cohort, it cannot be ruled out that alcohol consumption is linked or associated with greater social interaction and a sense of well-being, a fact that may have encouraged the sense of well-being in the practice of physical exercise in the participants of the current study during the pandemic.

Another important point observed in this study was the prevalence of interval training, in which the most prevalent physical exercise was aerobic activity. In the two years of the COVID-19 pandemic period, most adults were not affiliated with clubs and training associations, and did not receive guidance from a specialist for their training program. Second Washif et al. (2022) [5], team-based sports were generally more susceptible to changes than individual sports. The training habit was maintained between street running (outdoor) and the gym (indoor), with asphalt being the most common floor surface, and wearing traditional or minimalist sports shoes, but without guidance from a specialist. In addition, when guidance was provided, the prevailing professional was a physical educator or physiotherapist. According to Pagaduan et al., (2022) [17] the world class athletes were the least to use a self-designed training program, as they have received more assistance from their coaching staff.

Although the main concerns of COVID-19 focused on the cardiorespiratory system, COVID-19 can have pathological consequences in other organ systems, such as the musculoskeletal system, which can influence decision-making for the practice of physical exercise in athletes [27]. According to some authors, a pragmatic and monitored approach to the health of athletes should be adopted during the pandemic, especially in elite athletes and those who present symptoms and a diagnosis of COVID-19, with continuous monitoring by a health professional and the responsible technical team, since the practice of physical exercise or sport is a central component for maintaining a healthy lifestyle for athletes during the pandemic [27–29]. In the present study, it was observed that the vast majority of participants returned to the practice of physical exercise, with interval training for aerobic activities, however, without any access to a health professional or technician to monitor their physical and mental health. According to Alawna et al. (2020) [30], in a review study, after a diagnosis of COVID-19, all patients should be monitored by a physician or physiotherapist and directed to carry out an aerobic physical exercise program for 20 to 60 min, associated with muscle strength exercises.

Regarding the habit of physical exercise practices during the quarantine period for the prevention of COVID-19, a moderate training pace was observed by practicing adults, with the training environment being the street and the home (indoors), with a high prevalence of the morning period for the practice of functional exercises and weight training. The majority of participants reported not practicing sports during quarantine, however, of the 42.0% who chose to practice physical exercise, road running was the most prevalent (52.0%), with training being carried out individually and with care taken to follow the preventive measures of COVID-19, that is, with the use of a fabric and/or surgical mask, maintaining social distancing during the training. There is a general consensus in the literature that regular physical training of moderate volume (30–60 min, 3–5 days a week) and intensity (60–80% of maximum capacity) is associated with a general decrease in the risk of infection of the respiratory tract, especially during the pandemic [31, 32]. In the current study, it can be observed that the habit of practicing physical exercise among Brazilian adults was maintained during the pandemic, with functional exercises and weight training performed in outdoor (street) and indoor (indoors) locations, but without parameters of volume and intensity oriented by a health professional or sports coach.

There is no clear, evidence-based way to guide the return to exercise, but a prudent approach should be taken gradually, on an individual basis, and based on subjective exercise tolerance. Once a patient and/or athlete has been risk stratified and is free of COVID-19 symptoms for at least seven days, a phased, progressive exercise approach can be used to increase existing fitness level [30, 31]. It can be expected that people will be more out of breath to perform a certain physical exercise, especially after a period of COVID-19 or after inactivity. However, a degree of subjective judgment is needed to assess whether this is consistent with the patient’s activity and fitness level, and whether it is improving, in order to affect a graded progression in volume and intensity [31, 33]. In the current study, a high prevalence of COVID-19 symptoms was observed in 60.5% of physical exercise practitioners, with a positive diagnosis in 55% of the 304 participants. The majority reported having a consultation with a doctor and a diagnostic test was performed in 61% of cases, with the PCR test being the most prevalent test for confirming the diagnosis of COVID-19. In the vast majority of cases, the participant did not require hospitalization (95.0%) or assistance in the ICU (75.0%). Despite the large percentage of the presence of the disease, the majority did not obtain gradual monitoring for the practice of physical exercise, which is carried out predominantly without training guidance.

Another important point was the observation that during the COVID-19 pandemic, only 17% of the evaluated adults reported injuries, with patellofemoral syndrome making up 35.0% of the reports, limiting the functional activity of walking in 35.0%. Only 48% reported pain 24 h after training and 33% felt pain in the musculoskeletal system when returning to physical exercise practices. In this direction, no studies with reports of injuries and associated symptoms during the pandemic and in the return to physical exercise practice were found in the literature, making it difficult to compare with other evidence. However, according to one study performed in 2023 by Machado et al., [34], the quarantine period during the COVID-19 pandemic negatively affected the athlete’s perception about their training routine, since athletes reported a reduction in training hours and training intensity. Overall, the athletes reported that they were moderately to extremely anxious. Other evidence from the literature also supports that the COVID-19 pandemic influenced the behavior of runners, with increased training volume, decreased intensity and motivation, and a heightened injury risk, as well as reduced sleep quality and feelings of anxiety [12, 35, 36]. In the current study, we also observed a risk of injuries, since the training practice was not monitored by a professional or coach, and an increased feeling of anxiety reported by practitioners, a fact that can make them more vulnerable to inadequate training practices.

Regarding the feelings and emotions arising from the COVID-19 pandemic in the period of two consecutive years, as well as the reasons for practicing physical exercise, it was observed that anxiety was prevalent in 50.5% of adults. According to evidence from the literature, post-traumatic stress disorder, anxiety, and depression have been identified as a possible feature after diagnosis of COVID-19 [26, 31, 36]. In the current study, the vast majority of adults evaluated had a diagnosis of COVID-19 associated with a feeling of anxiety, which can be explained by the disease and conditions of the pandemic. Another interesting observation was the reason for practicing physical exercise, which was predominantly to maintain physical conditioning, followed by a feeling of pleasure, and weight loss. These reasons can be explained by dietary changes during the pandemic, in which intake of fast food, high carbohydrates, and saturated fat increased, resulting in worse physical fitness and depression conditions of the evaluated adults [23, 24, 26, 31, 36].

The limitation of this study was the lack of monitoring in the first year of the pandemic compared to the years 2021 and 2022, in which the quarantine periods were stricter and more restricted. Another important limitation was that the variables were not evaluated before the pandemic for a possible comparative effect before and after COVID-19. Future cohort studies comparing the first year of the pandemic to the present moment would be of great value for better understanding of physical exercise habits during the COVID-19 pandemic.

## Conclusion

During the two years of the COVID-19 pandemic (2021 and 2022) that were marked by periods of lockdown, physical activities altered to emphasize running and strength training, with low reports of injuries and pain symptoms after exercising. However, the restrictions negatively affected the exercise behavior due to respiratory tract symptoms after COVID-19 and a reduction in training intensity, this being without any technical supervision. In addition, the adults reported the use of a fabric or surgical mask for the prevention of COVID-19, and increased feelings of anxiety. The reasons for practicing physical exercise reported were physical conditioning, a feeling of pleasure, and weight loss.

## Electronic supplementary material

Below is the link to the electronic supplementary material.

Supplementary Material 1

## Data Availability

The datasets used and/or analyzed during the current study are available from the corresponding author on reasonable request.

## Abbreviations

BMI: Body Mass Index
COVID-19: Coronavirus Disease 2019
ICU: Intensive care unit
IgA: Immunoglobulin A
IgG: Immunoglobulin G
IgM: Immunoglobulin M
RT-PCR: Using real time- polimerase chain reaction
SARS-CoV-2: Severe Acute Respiratory Syndrome Coronavirus 2
