# Supplementary material for: Exercise behavior, practice, injury, and symptoms of respiratory tract infection of 502 Brazilian adults during lockdown oscillations in two years (2021–2022) of the COVID-19 pandemic
Source: BMC Sports Sci Med Rehabil. 2023 Aug 1;15:94. doi: 10.1186/s13102-023-00701-8 (PMC10394914; doi:10.1186/s13102-023-00701-8)
Supplement: Supplementary file 1 — Supplementary Material 1 [file 13102_2023_701_MOESM1_ESM.pdf]

QUESTIONNAIRE

Questionnaire physical exercise during the COVID-19 pandemic (coronavirus disease 2019)

Step 1 Questions	Step 2 Questions	Step 3 Questions	Step 4 Questions
What's your gender (sex)?	Do you have a clinical diagnosis of hip osteoarthritis?	Have you had an injury in the last 12 months?	Did you have symptoms of COVID-19?
What is your state of residence?	Do you have a clinical diagnosis of knee osteoarthritis?	What was the clinical diagnosis of the injury from the last six months or currently?	Did you consult a doctor?
Do you have a chronic illness?	Do you currently have pain in your feet?	What was the location of this injury?	Did you test for COVID-19?
Do you consume any alcoholic beverages socially?	Do you feel more pain in?	Do you feel pain when you.?	What test was performed?
How often weekly?	What is the most predominant location of pain in the feet?	Do you currently have persistent pain or physical impairment?	Test result was?
Which beverage?	Do you currently have knee pain?	Do you stop running because of pain?	If you had COVID-19, did you need to be hospitalized?
Do you consider yourself a smoker?	Do you feel more pain in?	Injuries (current)?	Did you need to go to the intensive care unit (ICU)?
What type of physical activity practitioner do you classify yourself as?	What is the most predominant location of pain in the knees?	Do you have a current injury?	Have you had contact with COVID-19 in the last 6 months?
What type of exercise do you practice?	What is your pace of training?	Did you have pain when returning to exercises?	What feelings and emotions during COVID-19?
Do you alternate the intensity of physical activity during the week?	What was the predominant location of your training practice during quarantine?	Have you interrupted practice because of pain?	What reasons for practicing exercises?
Do you perform interval training?	What is your typical training start time?	Do you have pain after training?	
If the previous answer was yes, in what way the exercise in interval training?	Did you practice more than one physical activity during isolation?	Do you have pain 24 hours after training?	
Are you affiliated with any club or coaching advisor?	What other physical activity did you practice?		
Do you have a training program (schedule)?	Did you practice any other sports modality during social isolation?		
What is the ground surface used in your training?	If the previous answer was yes, which sport?		
What is the ground surface used in your training?	Was your training supervised by a professional?		
What is your predominant training location?	If the previous answer was yes, how was it supervised?		
Do you warm up with pre-training stretching?	How was your training performed?		
Do you cool down with post-training stretching?	Did you follow preventive measures against the coronavirus?		
Do you perform muscle resistance training?	Did you use a protective mask during physical activity?		
For your training practice, do you know if you have vitamin D deficiency?	If the previous answer was yes, which type of mask did you use predominantly?		
What type of footwear do you use most during physical activity?	Did you maintain social distancing (1.5m) during physical activity?		
Did you receive guidance on your shoes for physical activity?			
If the previous answer is yes, which professional guided you?			
Do you use any bandages in training practice?			
